# An Interactive-Technology Health Behavior Promotion Program for Heart Failure Patients: A Pilot Study of Experiences and Needs of Patients and Nurses in the Hospital Setting

**DOI:** 10.2196/resprot.3188

**Published:** 2014-06-19

**Authors:** Rony Oosterom-Calo, Tineke A Abma, Merel A Visse, Wim Stut, Saskia J te Velde, Johannes Brug

**Affiliations:** ^1^Philips ResearchBriarcliff Manor, NYUnited States; ^2^EMGO Institute for Health and Care Research and the Department of Epidemiology and BiostatisticsVU University Medical CenterAmsterdamNetherlands; ^3^EMGO Institute for Health and Care Research and the Department of Medical HumanitiesVU University Medical CenterAmsterdamNetherlands; ^4^University of Humanistic StudiesEthics of CareUtrechtNetherlands; ^5^Philips ResearchEindhovenNetherlands

**Keywords:** heart failure, health promotion, interactive-technology, hospital, user needs, patients, nurses, physical activity, medication adherence

## Abstract

**Background:**

Heart failure (HF) is a chronic condition, prevalent especially among older people, characterized by acute episodes leading to hospitalization. To promote HF patients’ engagement in physical activity (PA) and adherence to medication, we developed Motivate4Change: a new interactive, information and communication technology (ICT)-based health promotion program for delivery in the hospital. The development of this program was guided by the Intervention Mapping protocol for the planning of health promotion programs. The users of Motivate4Change were defined as hospitalized HF patients and hospital nurses involved in HF patient education.

**Objective:**

Two aims were addressed. First, to explore the use of interactive technology in the hospital setting and second, to evaluate user needs in order to incorporate them in Motivate4Change.

**Methods:**

Participant observations at a hospital in the United Kingdom and semistructured interviews were conducted with hospitalized HF patients and HF nurses following their completion of Motivate4Change. Interviews were recorded, transcribed, and analyzed according to a thematic coding approach.

**Results:**

Seven patients and 3 nurses completed Motivate4Change and were interviewed. Results demonstrated that patient needs included empathic and contextual content, interactive learning, and support from others, including nurses and family members. The nurse needs included integration in current educational practices and finding opportunities for provision of the program.

**Conclusions:**

The current work provides insight into user needs regarding an interactive-technology health promotion program for implementation in the hospital setting, such as Motivate4Change.

## Introduction

Heart failure (HF) is a chronic cardiac condition prevalent especially among older people, characterized by acute episodes leading to hospitalization [1]. HF is usually irreversible but can be managed with medication and with nonpharmacological treatment, which includes a range of behaviors, such as dietary, physical activity (PA), self-management, and monitoring behaviors [2]. However, adherence rates to the behavioral treatment plans are suboptimal [3], which has been linked to adverse clinical outcomes [4]. There is a need for effective programs that promote the adherence of HF patients to behavioral treatment.

**Figure 1:**
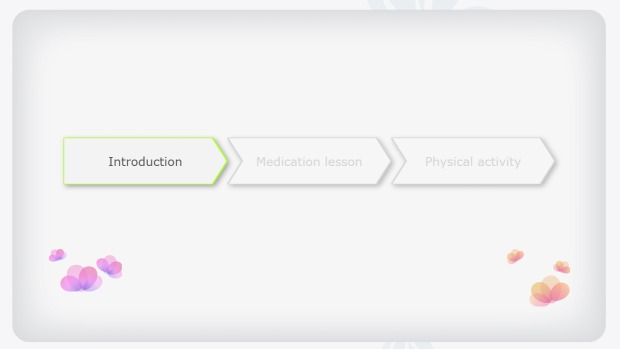
Screenshot of the main menu from Motivate4Change.

To promote engagement in PA and adherence to medication among patients with HF, we developed Motivate4Change: a new interactive health promotion program for delivery in the hospital (Figure 1). Patients with HF are often educated regarding self-management by HF nurses during hospitalization, and Motivate4Change was developed to assist nurses in the delivery of the education, as well as to optimize the promotion of self-management health behaviors among patients. The development of this program was guided by the Intervention Mapping protocol [5,6] for the planning of health promotion programs. Using this protocol, health promotion program planners can integrate evidence and theory in a structured manner in the planning of health promotion programs.

A user-centered design approach was followed in the development of the program, to incorporate user perspectives, needs, and preferences. When users are not involved, they often refrain from using a system [7]. If a program is poorly or incompletely implemented in the intended setting, or fails to reach a sufficient proportion of the target group, it may lack success [8]. The users of Motivate4Change were defined as hospitalized HF patients and hospital nurses involved in HF patient education and health promotion.

Motivate4Change was designed to educate hospitalized HF patients about PA and medication adherence, and motivate them to become more adherent to these behaviors after hospital discharge. The aim of the research is to formatively evaluate the use and experiences of patients and nurses with the program and its implementation in the hospital setting, in order to incorporate the results in a next version of Motivate4Change.

Motivate4Change consists of an introduction module, a medication adherence module, and a PA module. Within each module there are four components: (1) an introduction, (2) a list of take away messages (the key messages patients should remember from the module), (3) a video with information on medication adherence and PA that shows a “typical” HF patient, John, living his daily life and dealing with difficulties related to medication adherence and PA, and (4) assessments and feedback. Specifically, in the medication adherence module, an assessment of the patient’s knowledge on how to take medications appears first. Patients can answer the multiple choice questions by clicking a button on the tablet’s touch screen. Next, an assessment of barriers to medication adherence is presented. This assessment includes barriers of HF patients to taking medications. Finally, an assessment of beliefs about medications is presented. Next, in the PA module, an assessment of the patient’s knowledge on how to perform PA is firstly presented, followed by an assessment of barriers to PA performance. Based on their answer to each question in the assessments, patients receive tailored feedback messages.

Previous qualitative studies have been published about perspectives and experiences of older people with health technology [9,10]. One study, for example, explored reactions and perceptions to home monitoring systems of cognitive and physical health [9], and found that older people can accept home monitoring technology, if they perceive it as valuable. In addition, qualitative studies have been published about perspectives and experiences of health technology and Web-based health applications of patients with various health conditions [11-14] including HF [15]. For example, one study showed that stroke patients found an Internet portal with information on stroke easy to use and valuable [13]. These studies show that older patients can accept technology for health promotion and management.

However, few studies have explored patient experiences with information and communication technology (ICT) in the hospital setting. There are aspects of the hospital setting that may have an impact on the experiences of patients. For instance, the patient’s physical and emotional state when in the hospital [16] may influence their experiences with the program, which may influence its efficacy level. Therefore, the current work is an important addition to our understanding of the experiences with new technology-based health promotion programs for older patients in a setting that has not yet been explored, but may be used more in the future. Qualitative research is an effective approach for gaining in-depth information about topics on which there has been limited prior research [9].

When there is a lack of knowledge on a particular topic, grounded theory is a particularly useful framework to adopt as the research framework [17,18]. According to grounded theory, behavior is influenced by the context in which it takes place. In the current research, the context of the hospital setting will be studied, and the meaning of experiences and behaviors of patients and nurses in this context will be interpreted.

In sum, two aims were addressed in the current work. First, to explore the use of, and experiences with, interactive technology used in health promotion delivered in the hospital setting, and second, to evaluate user needs in order to incorporate them in Motivate4Change.

## Methods

### Sampling

A convenience sample of 7 patients and 3 nurses was selected from an academic hospital in the United Kingdom, which has a total of 610 beds including 42 on the cardiology ward. To select participant patients, the following inclusion criteria were applied: (1) they were hospitalized for HF, (2) they were able, cognitively and physically, to use a tablet and read the information provided in the Motivate4Change program, and (3) they did not have a medical condition (eg, an infection) that could infect the researcher. Patients awaiting valve surgery were excluded, because the behavioral recommendations for these patients may be different than for the rest of patients, depending on the outcome of the surgery. An attempt was made at achieving maximum variation, in terms of age, sex, and experience with ICT. However, the sample was restricted to patients that were hospitalized for HF at recruitment period of the study. Participant nurses were recruited based on their involvement in HF patient education and behavior change promotion during hospitalization. Only nurses who have HF patient education tasks in their role were included.

### Recruitment

Recruitment to the study took place during two phases in 2012. Each phase was 1 work-week long. In each phase, to select patients meeting the inclusion criteria, a nurse reviewed the list of patients on a daily basis, and selected patients she believed met the criteria. She then approached the patients at their hospital bed, described the study, provided a patient information sheet that described the study in detail, and asked for their participation. If a patient agreed to participate, a suitable time was discussed. All nurses who met the inclusion criteria were contacted by email and asked to participate by the researcher. If they agreed, a suitable time was discussed.

### Procedure

The researcher (first author) approached participant patients at the agreed time by their hospital bed, or at a separate room on the ward, depending on the preference of the participant. The researcher asked each participant to sign an informed consent form, and then provided them with Motivate4Change on a tablet and a brief explanation on how to use the program. Each participant then started the program, while the researcher sat on the ward, in the vicinity of the participant, and waited for each patient to go through the whole program. If participants had questions, these were answered by the researcher. After they had completed Motivate4Change, the researcher turned on an audio recording device and interviewed the patients according to the predefined, semistructured interview guide. Interviews lasted between 10 and 40 minutes (Table 1). The interview guide explored participants’ expectations of Motivate4Change, personal assessments of the program, experiences receiving Motivate4Change on interactive technology, and possible behavior change as a result of using the program.

Observations were made by the researcher during the periods of time that participants were completing Motivate4Change, while sitting in the ward. These periods were approximately 45 minutes per patient. An observation protocol was used. Items included possibilities and obstacles for providing a health promotion tablet on the cardiology ward and potential user needs expressed nonverbally. Extensive field notes were taken during the observations periods, and if any questions arose, or if any issues required clarification, these were discussed by the researcher with the participating nurses.

The interviews with nurses were conducted at office rooms at the hospital. First, the researcher gave a brief explanation about the program and the study, then nurses were provided with the tablet and completed the Motivate4Change program, and finally structured interviews took place using a semistructured interview guide, and were recorded with an audio recording device. The interview guide explored nurses’ expectations and opinions regarding use of interactive technology as a tool for health behavior promotion in the hospital, possible fit of Motivate4Change in their patient education-related workflow, and possible impact on patients.

**Table 1:** Participant characteristics.

Participant number/ interview length, in minutes^a^	Age	Experience with ICT^b^: PCs^c^, smartphones, and tablets
Patient 1/20	58	None
Patient 2/25	75	None
Patient 3/20	76	None
Patient 4/30	71	None
Patient 5/40	75	Experience with laptop, needs help with internet
Patient 6/10	37	Experience with PC’s^c^, tablets and smartphones
Patient 7/40	64	Experience with tablets and smartphones but needs help using them
Nurse 1/15	48	Experience with PC’s^c^, smartphones and tablets
Nurse 2/20	36	Limited experiences with PC’s^c^, smartphones and tablets
Nurse 3/30	45+	Experience with PC’s^c^ and smartphones, limited experience with tablets

^a^Rounded to the nearest whole minute.

^b^Information and communication technology.

^c^Personal computers.

### Analysis

Data was analyzed according to the grounded theory approach, which postulates that data collection and analysis are interrelated. The analysis of the first interviews directs the analysis of the next interviews. Therefore, the results of a study that uses this approach are achieved through an iterative process of collecting and analyzing data [18]. In order not to miss anything important, it is crucial to analyze all information and see if any new information appears that was not prospectively considered. An inductive approach to the analysis will be adopted, whereby explanations and patterns will be sought with a bottom-up approach [18].

The audio recordings of the patient and nurse interviews were transcribed. The patient interviews were analyzed separately from the nurse interviews in order to assess both perspectives. The text of the first 3 patient interviews was coded separately by 2 researchers, in order to maximize the quality of the analysis [18]. Coding the text was achieved by assigning codes to chunks of data in order to label and organize the data. First, codes were identified and created. Then, data was assigned to codes. The emerging themes were captured. Finally, codes were sorted into categories. In this stage a coding framework was developed. Codes were grouped into categories in an iterative process. This coding process was first completed for the interview transcript separately (open coding), then a process of axial coding was initiated, were material from the various transcripts was related and compared. Also, the texts from the field notes were analyzed by assigning codes to chunks of data and grouping the codes into categories, and finally capturing the emerging themes. Topics from patient interviews and from the observations were informally checked with the nurses.

## Results

### Patient and Nurse Participation

Seven patients completed Motivate4Change and were interviewed. The age range of patients was 37-76 (Table 1). All patients were male and had been diagnosed with HF before the current hospitalization. Patients had varying levels of experience with ICT. Twelve patients met the inclusion criteria and 4 refused to participate. One of the 8 patients who agreed to participate was sleeping when the researcher arrived at the scheduled time, and was discharged soon thereafter. Reasons for nonparticipation included participating in other studies and not wanting to participate in more studies during hospitalization and feeling upset following the recent HF diagnosis.

The analysis of all data revealed two general themes, patients’ and nurses’ needs. The theme “patients’ needs” was divided into three subthemes, including contextual and empathic content, interactive learning, and support from others. The theme “nurses’ needs” was divided into two subthemes, including integration in current educational practices and finding opportunities for provision of the program.

### Patients’ Needs

#### Contextual and Empathic Content

Participating patients have been living with HF before using the Motivate4Change program, and they viewed the information in Motivate4Change in light of their own experiences with HF management. They viewed the content as empathic as it acknowledged their emotions and own personal experiences, and contextual, since it addressed the context of their life as HF patients. Patients felt their personal situations were acknowledged in the videos and with the assessment questions and feedback, and this seemed to encourage them that PA is relevant and possible for them. For example, one patient remarked that “he [John from the video] wasn’t an athlete. He was just an ordinary chap. He was doing all sorts of things” [Patient 04], and concluded that he can start being more active after his discharge from the hospital. In addition, various pieces of information from Motivate4Change were recognized by patients based on their own physical abilities, and this acknowledgement of their abilities made them feel encouraged to perform PA in the future. A patient that has not been doing enough PA, and has been experiencing limitations that make it difficult to be physically active, recalled from the Motivate4Change content that he can build up his aerobic capacity by performing a little more activity on a daily basis. Another patient, that has been very active, recalled that he should pace himself and spread the activities throughout the day. Nurses also appreciated the contextuality of the videos and the fact that the video provides a concrete example of a HF patient engaging in the behaviors in daily life situations “to see potentially what a patient can do” [Nurse 2].

The importance of contextuality was demonstrated also in discussions about the medication adherence content. Most patients did not view this content as acknowledging their personal situation, since they indicated they are adherent to their medications and viewed Motivate4Change as more relevant for patients who are not: “Well, I think it would benefit some people, because I know some people think that they are alright and they can stick the day with one tablet” [Patient 03]. Regardless, some patients did recognize that some difficulties to taking their medications were acknowledged. For example, one patient remarked, “That bloke going out and not taking his diuretic. I know what it is like”, referring to fluid retention that results from skipping a diuretic dose. Although most appreciated having a repetition of information regarding medication adherence, patients appreciated the information on medication adherence to a lesser extent than that on PA, due to the fact that it was not perceived as personally relevant.

The empathic tone of the program, which included acknowledgement of possible difficulties and emotions, evoked feelings of safety and reduced insecurity and fear. Patients indicated that HF patients can be panicked, and that Motivate4Change can help with this and help people understand they can still do things:

You know, people tend to […] panic! They do! I’ve seen them, seen lots of them! […] you say it in a nice way, you see what I mean [ ...] because people are so… they panic you see. Heart failure!? Oh! Panic, panic, panic! […]People are frightened. But that [Motivate4Change] is sensible. It’s telling to keep going [...]. So that’s right, that’s a good idea then. […] you can still do this, you can still do that, you can still do more things […] and learn how to go back to the way you were. That’s what you’re doing with that [Motivate4Change], isn’t it?

Patient 07

Notably, not all patients felt acknowledged by the program. One patient, who was younger than a typical HF patient (37-years old) did not perceiveMotivate4Change as relevant for him because he thought it addresses only older people. He mentioned that John, the character from the video, is an older man. Another patient, who receives home care including medication support and a physiotherapist to help with PA, did not perceive that Motivate4Change was relevant for him since his situation was not acknowledged. Likewise, participating patients commented that Motivate4Change would not be relevant for patients with cognitive and physical limitations, or depression.

Well, you may find an old lady that does not understand anything very much. And that gentleman there, it would be a waste of time to show him this, they can’t even get him to read, he would not be into that.

Patient 05

#### Interactive Learning With ICT

The patients expressed their preferences for learning in an interactive manner. This was in comparison to being “told” what to do with traditional written materials.

I think that [Motivate4Change] is probably more useful to people than, you know, some of the books […] because it interacts with people, and the book doesn’t ask you a question. It’s just telling the things. When it’s asking a question, you can think about it. If you get the answer wrong it puts you right. And if you look at the pamphlets, all they do is tell this, that, and the other, it’s like reading a novel, in a sense

Patient 05

They perceived that interactive learning better addresses their learning needs. It provided information that is relevant for them and included less information.

The trouble [with the booklets] is that they try to give people complete information. It’s information overload to some. […]. And some of the other pamphlets overlap quite a lot. […] So this [Motivate4Change] provides a better picture of what you need to do

Patient 05

Nurses appreciated the interactivity because of its potential to facilitate learning “they [patients] can get both answers, in case it is not clear in their mind what the right answer is” [Nurse 02]. Nurses also related the program to digital video discs (DVDs) provided at hospitals in the United Kingdom to post myocardial infarction (MI) patients. They remarked that Motivate4Change could work in a similar way, but they appreciated the interactive aspect in Motivate4Change, which is not available in DVDs.

MI patients have a lot of different DVDs, which they can watch and use […] I think for the heart failure patients they need more things. And different tools to help them […]. So, yeah, I think things like this [Motivate4Change] are great. I do really like the format.

Nurse 02

Nurses also made clear that a prerequisite to the provision of an interactive technology program is simplicity. They also indicated that Motivate4Change is simple:

you only have to switch it on and it loads on- I think it’s really easy. […] You have only got one button to press

Nurse 02

Terms such as “straightforward”, “not too complicated”, “quite easy to show to people”, “not too difficult for the patients” [Nurse 03], “easy to use, really easy to use”, “user-friendly” [Nurse 02] were brought up. Nurses considered simplicity to be an advantage of the program.

It was interesting that although most of the interviewed patients had a positive or neutral attitude toward the use of ICT as a means to receive health promotion information, some patients expressed a general negative attitude toward ICT. One patient, for example, commented that he has “seen some people become slaves to them [computers]” and associated computers with “not doing things” [Patient 04]. Notably, some patients viewed nurse education sessions as more interactive than ICT-based education:

she gives feedback when you ask a question. She answers everything and she is there for a lot longer

Patient 06

One patient commented that he already received information from nurses and doctors. He felt that there was no need to also get this from a computer: “you don’t need a computer to tell you to walk” [Patient 02].

The nurses, however, thought Motivate4Change can facilitate the interaction that patients have with them in the educational sessions, and thereby make the sessions with them more interactive. They indicated that patients may not think of all of their questions during the face-to-face education sessions, and by using Motivate4Change patients can become triggered to ask additional questions in the sessions. The nurses indicated this is especially relevant for newly-diagnosed patients, who have little knowledge about HF and therefore no pre-existing questions to ask.

It was observed by the researcher that all patients read all of the information. When asked why he read everything in the program, one patient remarked,

It looked interesting. When you go further into that, it was more interesting

Patient 01

Which indicates that something compelled him to continue to completion. Interestingly, patients read the information regardless of whether the attitude to learning that they expressed was positive or indifferent. Specifically, some patients expressed a positive attitude toward learning:

Oh I am the one that ought to read everything. Not everybody does! It’s just me […] Yes, I do try to learn.

Patient 07

Other patients were indifferent to learning about HF management. They even preferred to refrain from learning-related activities:

I think things like this anyways, you just look at them if you want to look at them. You know what I mean? I’ve always tried to avoid this business

Patient 06

When the researcher asked if he read the booklet that was provided to him in the past by the nurse, the patient remarked: “not all of it, but the bits that I was interested in, well, not interested in, that seemed relevant” [Patient 06], indicating that booklets were not completely read in the way the content in Motivate4Change was. This patient further indicated although he did not appreciate the content of Motivate4Change (as discussed above) he did appreciate the interactivity, and even made suggestions for additional features. The reason for completion seemed to be, therefore, the interactive aspect, which was appreciated even when the content was not.

#### Support From Others

Patients would need some support from nurses to engage in the program. First, they would require an explanation regarding the meaning of the program and to how to use it. All patients indicated that Motivate4Change was easy for them to use, including the patients who expressed negative attitudes toward ICT. Indeed, it was observed that all patients could use the program, although they sometimes leaned on the tablet with their hands, which caused usability issues. Some patients demonstrated insecurity by asking the researcher questions regarding how to navigate, while, in fact, clicking on the correct buttons. This might indicate that some support would be needed to reduce the insecurity.

Also, some patients remarked that it is difficult to complete the program when lying in bed, since it can cause them to lose their attention. Indeed, it was observed that patients could better interact with, and seemed more attentive, when performing the program while sitting in the chair, which was available at every bedside. Therefore, patients may need some support getting out of bed and sitting on the bedside chair, or walking to the common room on the ward.

The nurses indicated that family members could also use the program, and thereby support patients, both during the program use, as well as after discharge from the hospital. “I think families could get quite a lot of benefit from it, because they come to visit somebody […] and want to know what’s happening, how easy is it for them to flip through something like this?” [Nurse 01]. They specified that some family members visit only during the late visiting hours and do not get to meet with the nurse, and Motivate4Change can be used to inform them about HF self-management.

Finally, patients would have to know that they have been diagnosed with HF and what HF is, and be ready to receive information regarding HF self-management, before receiving Motivate4Change, because, according to nurses, it could be psychologically difficult to deal with the diagnosis. Therefore, before providing the program, nurses would need to assess if patients are aware that they have HF and are ready for receiving information about HF self-management.

### Nurses’ Needs

#### Integration in Current Educational Practices

Motivate4Change was placed by patients and nurses in the context of past and future educational encounters. They discussed booklets, pamphlets, and nurse education sessions as learning materials they have previously received (patients) or used in their education sessions (nurses). Motivate4Change was viewed as a welcome addition to these materials, since it can reinforce their existing knowledge:

I felt that (Motivate4Change) reinforced what I’ve read, which is a good idea, because if somebody talks to you for a while you’re not going to remember all of it. If you see it again in a book, you think “oh yes I remember that” – if you see it in a different format altogether it’s another cue to your memory so it’s a good idea.

Patient 05

Since nurses are those that provide patients with education, including the various educational materials, they discussed the need for Motivate4Change to be integrated with current educational practices. They expressed their opinion that the program could be most efficacious in conjunction with other learning materials (leaflets, booklets, face-to-face sessions), which are typically already provided to patients in current care practices.

In addition, they discussed the efficacy of home-based education provided by the community nurse. This education is provided in the context of the setting where patients actually perform PA and take their medications. Nurses recommended that this type of education would be provided in conjunction with Motivate4Change to reinforce it in a real-life setting. Patients who have already been receiving education at home viewed Motivate4Change also in relation to this type of education, and expressed their desire to keep their contact with the community nurse:

if I’m poorly she knows whom to phone […] yeah I still need her to come […] she checks me out, feet, breathing, everything. She makes sure my lungs are clear. She’s a good nurse.

Patient 03

#### Finding Opportunities for the Provision of the Program

Finding a suitable opportunity for nurses to provide, and thereafter for patients to complete, Motivate4Change is an issue of importance to nurses. One of the nurses described how she would introduce the program:

You would discuss the heart failure, the condition, what’s it about. Then, a little bit down the line, you would introduce that [Motivate4Change] and leave it with the patient and while you’re doing that you could go off and start with another patient the basic introduction to HF and so on.

Nurse 02

Most patients took approximately 1 hour to complete the whole program, including both modules, but 1 patient took about 30 minutes, and another requested a break between the two modules. A few patients discussed that they would “need their time with it”. According to the nurses it would be necessary for patients to be able to go through Motivate4Change on their own, as the nurse would not be able to do this with all patients individually due to time constraints. It was observed, and corroborated with information from nurses, that due to medical procedures, physician rounds, visiting hours and meal times, and the fluctuating symptoms patients may experience in the hospital, it may be challenging to find an uninterrupted period of time to complete the program during hospitalization. The researcher observed that, during the recruitment process, it was often difficult to find a time slot to administer Motivate4Change. Some patients gave their informed consent, but felt too unwell at the time the researcher arrived to administer the program, or were engaged with one of the aforementioned activities. The HF nurses suggested letting the ward nurses provide the program outside of their own working hours. This may maximize the time being spent on education, since patients would be able to become educated also when the nurses are away. If tablets were to be left on the ward, however, their placement would need to be considered. Although leaving it on the bedside table may be possible, it can fall or be stolen. In addition, it was observed that bedside tables are filled with items, and there is limited space available on them.

The nurses indicated that no added work would result if they would be using Motivate4Change as an educational tool. One of the nurses indicated that the time nurses would need to spend on training patients before using Motivate4Change would be minimal.

In sum, it was found that, regarding Motivate4Change, a health promotion program delivered on ICT in the hospital setting, patients appreciate empathic content, which acknowledges their personal situation. Patients appreciate receiving an interactive program and they are able to use such a program, even when they have limited experience with ICT. Finally, when completing a health promotion program delivered on ICT in the hospital setting, patients require some support from others, including nurses and possibly family members. Nurses require an integration of a new program in current educational practices and tools. Finding an opportunity to deliver the program to patients is a challenge for nurses, but possible ways to overcome this exist, including making the program available to patients also when HF nurses are not present on the ward.

## Discussion

### Aims of the Study

Two aims were addressed in the current work. First, the possible use of interactive technology-based health promotion programs in the hospital setting was explored. This investigation is especially interesting because education and health promotion via interactive technology has not been previously explored in the hospital setting, and therefore information on users’ experiences is scarce. Second, we aimed to evaluate user needs in order to incorporate them in Motivate4Change. In order to obtain the most accurate information regarding user needs in relation to the environment intended for the program’s use, we conducted the study in a hospital setting. Assessing user needs is a vital step in the development of innovative technologies, and if this step is not taken, the new technology may not be accepted, and therefore not taken up by the intended user population [7]. As such, researchers have called for exploration of user needs and their integration in the development of applications [19,20].

### Discussion of the Findings

Acknowledgement of patients’ own situation, via contextual content, was a central need for patients. By acknowledging their experiences with HF self-management, as well as the difficulties they experience, and by demonstrating how, regardless of these difficulties, health behaviors can be performed, patients felt empowered to perform the behaviors, and this empowerment motivated patients to perform them. Self-efficacy, or the perception that one is able to perform a behavior, is one of the central constructs of Social Cognitive Theory (SCT) [21]. Based on previous research, behavior change interventions for patients with chronic diseases that focus on enhancing self-efficacy are promising [22].

When patients did not perceive that their personal situation was acknowledged, as was the case with a younger patient, they were less engaged in the program and expressed a lack of appreciation of it. It is therefore important that all individuals feel acknowledged, even those that are less “typical,” although it may be difficult to achieve this. It remains to be seen if Motivate4Change is perceived as personally relevant for female and newly diagnosed patients, since all participants were male and have been living with HF previously.

Common difficulties experienced by HF patients were acknowledged in Motivate4Change by including a video with a typical HF patient as a role model. According to SCT role modeling is a strategy to increase self-efficacy, whereby seeing similar others perform certain behaviors leads to expectations about one’s own efficacy in performing these behaviors [21]. Therefore, acknowledgement through a role model, as is done in Motivate4Change, is also expected to lead to an increased level of self-efficacy, and thereby behavior change. In addition, patients’ personal situations were acknowledged by providing tailored feedback messages. Tailoring of health communications is a behavior change strategy in which information is provided to individuals based on the unique characteristics of those individuals [23,24]. To achieve this, people are assessed regarding characteristics of interest [23] and need to actively provide input. This approach has been found to be effective in changing health behaviors [24-26]. In addition, it requires the patients to actively interact with the program, an aspect of Motivate4Change that patients and nurses particularly appreciated.

Interactive learning was a second need for patients. Both patients and nurses thought that the interactivity aspect of Motivate4Change can facilitate learning. It should be pointed out that the interactivity in Motivate4Change was very simple and included either pressing only one button (eg, “next” to navigate to the next page) or choosing one out of two buttons to press (eg, selecting “true” or “false” to answer a question). Importantly, one of the strengths of Motivate4Change, according to nurses, was its simplicity. It was stressed that a program delivered via ICT to HF patients should be easy for patients to use.

It remains to be seen, however, if simplicity is sufficient for patients’ use of the program. In the current pilot study, all patients used the program; however, it is unclear if they would have used it if the researcher was not present. Although most patients expressed positive attitudes toward using ICT during their hospital stay, and very few usability problems were observed, some insecurity regarding their ability to use Motivate4Change was expressed by patients and also observed by the researcher. Previous research on the use of telerehabilitation by chronic pain patients demonstrated that although patients may perceive benefits to using technology, insecurity in their abilities to use it may be a barrier [27]. Further research is necessary on patients’ feelings of insecurity with the use of technology, the sources of this insecurity, and its potential effects on their actual use of the technologies, in order to address this issue in future programs.

A third need for patients was support in the use of the program, especially regarding the initiation of use. This support would be provided by HF nurses, since they are in charge of patient education, and it is therefore important that they appreciate Motivate4Change. The nurses interviewed in the current study welcomed Motivate4Change as a possible addition to their current educational tools. Their needs included integrating Motivate4Change in their current educational practices. Nurses discussed that they would potentially use Motivate4Change in their current educational routine and how they would integrate it in current educational practices.

It was discussed by nurses that a good option would be to also involve family members in the program, which could be an additional source of support to patients. The role of family members is central in patient self-management [28], and as such they could also potentially be involved in the process of patient motivation and activation.

Nurses viewed challenges regarding opportunities to deliver the program to patients. Possible ways to overcome this were brought up, including making the program available to patients also when nurses are not present at the hospital, so they can use it on their own. Family members may be instrumental if this option were to be pursued and their needs should therefore be explored in future research. It was observed that leaving a tablet with patients on the ward presents challenges, including location to place the tablet and safety issues. It is therefore advisable to create a solution that takes into account also the physical environment in the hospital.

Some patients discussed the importance of their connection with the nurses. They expressed a desire to keep the human contact and the ongoing support provided by the nurses. This is in line with previous research [29], which showed that some patients refused participation in a telehealth and telecare trial in part due to fear of losing their relationships with their care providers. Patients also regarded the education provided by nurses to be more interactive than the education provided by Motivate4Change. Interpersonal health communication, such as face-to-face educational sessions provided by nurses, is the most individualized form of health communication [23]. However, it also requires the highest level of assessment of individuals [23], which could mean it is more labor-intensive, and therefore more costly, than tailored health communication. It remains to be seen how to combine education and health promotion strategies, which are ICT-based with those provided by health care providers in a manner that is appreciated by most patients.

### Strengths and Limitations

The current work provides insight into possible acceptance and use of Motivate4Change. Scant information about the acceptance and use by patients and nurses of ICT-based health promotion programs in the hospital setting is available to date. Qualitative research methods are well suited to address this topic, since they allow exploring issues on which there is little information [18] because in the context of a qualitative study it is possible to ask patients to describe their experiences and provide feedback, rather than to use a predefined questionnaire, requiring a priori assumptions on behalf of the researcher [18]. In addition, the current study was conducted in the natural environment where usage of the program would occur. This can lead to rich information and reveal aspects that would not be uncovered if the researcher does not take part in the environment to truly understand it [18]. Moreover, three sources of information were used to reach conclusions, including interviews with patients and nurses, and observations. In this manner, a higher level of confidence in the results is reached.

However, the sample size was relatively small and the research was conducted in one specific setting, making it difficult to generalize the results. Since the recruitment of potential participants was limited to patients hospitalized during the recruitment period, the sample included only male patients that were experienced with HF. In addition, it is difficult to make concrete conclusions regarding the effects of Motivate4Change. An additional investigation, in the form of a clinical trial with quantitative results, is necessary to investigate the effects of the program on patients’ behavior and on clinical outcomes.

## Abbreviations

DVD: digital video disc
HF: heart failure
ICT: information and communication technology
MI: myocardial infarction
PA: physical activity
PC: personal computer
SCT: social cognitive theory

## References

[1] Kannel WB (2000). Incidence and epidemiology of heart failure. Heart Fail Rev.

[2] McMurray J, Adamopoulos S, Anker S, Auricchio A, Böhm M, Dickstein K, Falk V, Filippatos G, Fonseca C, Gomez-Sanchez MA, Jaarsma T, Køber L, Lip GY, Maggioni AP, Parkhomenko A, Pieske BM, Popescu BA, Rønnevik PK, Rutten FH, Schwitter J, Seferovic P, Stepinska J, Trindade PT, Voors AA, Zannad F, Zeiher A, ESC Committee for Practice Guidelines (2012). ESC Guidelines for the diagnosis and treatment of acute and chronic heart failure 2012: the Task Force for the Diagnosis and Treatment of Acute and Chronic Heart Failure 2012 of the European Society of Cardiology. Developed in collaboration with the Heart Failure Association (HFA) of the ESC. Eur Heart J.

[3] van der Wal MH, Jaarsma T (2008). Adherence in heart failure in the elderly: problem and possible solutions. Int J Cardiol.

[4] van der Wal MH, van Veldhuisen DJ, Veeger N, Rutten F, Jaarsma T (2010). Compliance with non-pharmacological recommendations and outcome in heart failure patients. Eur Heart J.

[5] Parcel GS, Kok G, Gottlieb NH (2006). Planning health promotion programs: an intervention mapping approach.

[6] Kok G, Schaalma H, Ruiter RA, van Empelen P, Brug J (2004). Intervention mapping: protocol for applying health psychology theory to prevention programmes. J Health Psychol.

[7] Johnson CM, Johnson TR, Zhang J (2005). A user-centered framework for redesigning health care interfaces. J Biomed Inform.

[8] Saunders RP, Evans MH, Joshi P (2005). Developing a process-evaluation plan for assessing health promotion program implementation: a how-to guide. Health Promot Pract.

[9] Wild K, Boise L, Lundell J, Foucek A (2008). Unobtrusive in-home monitoring of cognitive and physical health: reactions and perceptions of older adults. J Appl Gerontol.

[10] Demiris G, Rantz M, Aud M, Marek K, Tyrer H, Skubic M, Hussam A (2004). Older adults' attitudes towards and perceptions of "smart home" technologies: a pilot study. Med Inform Internet Med.

[11] Gerhards SA, Abma TA, Arntz A, de Graaf LE, Evers SM, Huibers MJ, Widdershoven GA (2011). Improving adherence and effectiveness of computerised cognitive behavioural therapy without support for depression: a qualitative study on patient experiences. J Affect Disord.

[12] ten Napel-Schutz MC, Abma TA, Bamelis L, Arntz A (2011). Personality disorder patients’ perspectives on the introduction of imagery within schema therapy: a qualitative study of patients’ experiences. Cognitive and Behavioral Practice.

[13] Rochette A, Korner-Bitensky N, Tremblay V, Kloda L (2008). Stroke rehabilitation information for clients and families: assessing the quality of the StrokEngine-Family website. Disabil Rehabil.

[14] Nijland N, van Gemert-Pijnen J, Boer H, Steehouder M, Seydel ER (2008). Evaluation of internet-based technology for supporting self-care: problems encountered by patients and caregivers when using self-care applications. J Med Internet Res.

[15] Seto E, Leonard KJ, Cafazzo JA, Barnsley J, Masino C, Ross HJ (2012). Perceptions and experiences of heart failure patients and clinicians on the use of mobile phone-based telemonitoring. J Med Internet Res.

[16] Giuli C, Spazzafumo L, Sirolla C, Abbatecola AM, Lattanzio F, Postacchini D (2012). Social isolation risk factors in older hospitalized individuals. Arch Gerontol Geriatr.

[17] Corbin J, Strauss A (1990). Grounded theory research: procedures, canons, and evaluative criteria. Qual Sociol.

[18] Holloway I (2005). Qualitative research in health care: edited by Immy Holloway.

[19] Arnhold M, Quade M, Kirch W (2014). Mobile applications for diabetics: a systematic review and expert-based usability evaluation considering the special requirements of diabetes patients age 50 years or older. J Med Internet Res.

[20] Grindrod KA, Li M, Gates A (2014). Evaluating user perceptions of mobile medication management applications with older adults: a usability study. JMIR Mhealth Uhealth.

[21] Bandura A (1977). Self-efficacy: toward a unifying theory of behavioral change. Psychol Rev.

[22] Marks R, Allegrante JP, Lorig K (2005). A review and synthesis of research evidence for self-efficacy-enhancing interventions for reducing chronic disability: implications for health education practice (part II). Health Promot Pract.

[23] Kreuter M, Farrel D, Olevitch L, Brennen L (2000). Tailoring health messages: customizing communication with computer technology.

[24] Brug J, Oenema A (2003). Campbell M: past, present, and future of computer-tailored nutrition education. Am J Clin Nutr.

[25] Noar SM, Benac CN, Harris MS (2007). Does tailoring matter? Meta-analytic review of tailored print health behavior change interventions. Psychol Bull.

[26] Broekhuizen K, Kroeze W, van Poppel MN, Oenema A, Brug J (2012). A systematic review of randomized controlled trials on the effectiveness of computer-tailored physical activity and dietary behavior promotion programs: an update. Ann Behav Med.

[27] Cranen K, Drossaert CH, Brinkman ES, Braakman-Jansen AL, Ijzerman MJ, Vollenbroek-Hutten MM (2012). An exploration of chronic pain patients' perceptions of home telerehabilitation services. Health Expect.

[28] Fisher L, Weihs KL (2000). Can addressing family relationships improve outcomes in chronic disease? Report of the National Working Group on Family-Based Interventions in Chronic Disease. J Fam Pract.

[29] Sanders C, Rogers A, Bowen R, Bower P, Hirani S, Cartwright M, Fitzpatrick R, Knapp M, Barlow J, Hendy J, Chrysanthaki T, Bardsley M, Newman SP (2012). Exploring barriers to participation and adoption of telehealth and telecare within the Whole System Demonstrator trial: a qualitative study. BMC Health Serv Res.

